# tRF‐Glu‐TTC‐026 as novel diagnostic biomarkers for active tuberculosis and regulates intracellular survival of Mycobacterium tuberculosis in macrophages by regulating macrophage polarization

**DOI:** 10.1002/ctm2.1781

**Published:** 2024-07-23

**Authors:** Zikun Huang, Cuifen Xiong, Qing Luo, Juxiang Zhu, Yongqin Guo, Peng Fu, Haiyan Zhu, Jianqing Xu, Yang Guo, Yiping Peng, Cheng Qing, Junming Li, Aiping Le

**Affiliations:** ^1^ Department of Clinical Laboratory The First Affiliated Hospital Jiangxi Medical College Nanchang University Nanchang Jiangxi China; ^2^ Nanchang Key Laboratory of Diagnosis of Infectious Diseases Gaoxin Branch Of The First Affiliated Hospital, Jiangxi Medical College, Nanchang University Nanchang Jiangxi China; ^3^ Department of Tuberculosis Jiangxi Chest Hospital Nanchang Jiangxi China; ^4^ Department of Intensive Care Medicine Medical Center of Anesthesiology and Pain The First Affiliated Hospital Jiangxi Medical College Nanchang University Nanchang Jiangxi China; ^5^ Department of Blood Transfusion Key Laboratory of Jiangxi Province for Transfusion Medicine The First Affiliated Hospital Jiangxi Medical College Nanchang University Nanchang Jiangxi China

Dear Editor,

Despite efforts to control it, tuberculosis (TB) remains a significant challenge in the field of public health globally.^1^ Timely diagnosis of active TB is essential to control disease transmission.^2^ A recently discovered group of small non‐coding RNAs, namely transfer RNA‐derived small RNAs (tsRNAs), are present in high levels and exhibit stable expression in humans.^3^ Some studies have shown that tsRNAs can be used as disease diagnostic markers,^4^, ^5^ but its value in TB is still unclear.

A multi‐stage case–control study was developed to identify tsRNAs in peripheral blood mononuclear cells (PBMCs) as biomarkers for TB diagnosis. The study enrolled 200 TB patients, 141 latent TB infection (LTBI), 132 pneumonia (PN) and 150 healthy controls (HC) (Figure 1, Table S1). The study identified 128 tsRNAs with differing expression patterns, with 33 showing decreased levels and 95 showing increased levels (Figure 2A–C, Table S2).

**FIGURE 1:**
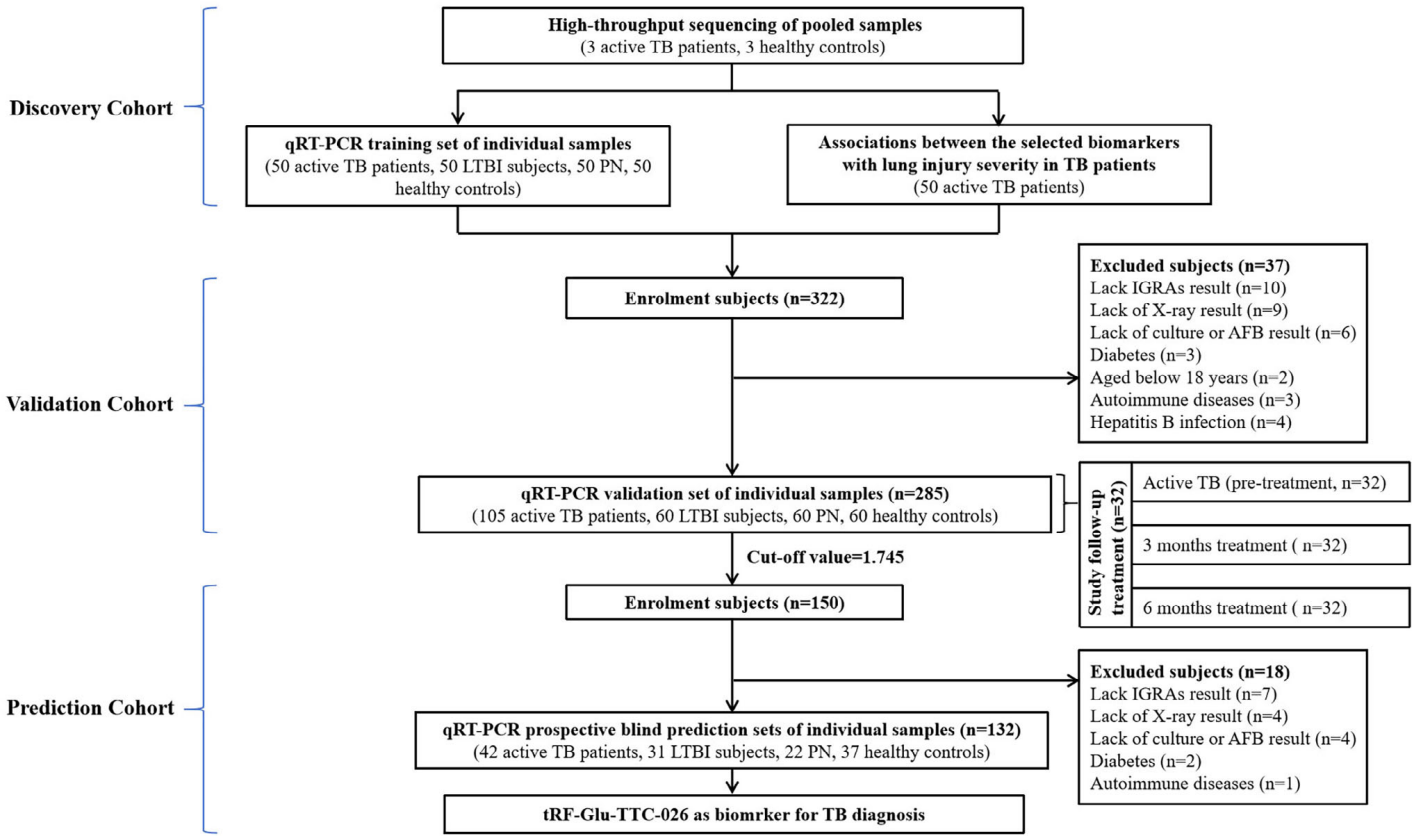
Study design and classification of study participants. LTBI, latent TB infection; PN, pneumonia; TB, Tuberculosis.

**FIGURE 2:**
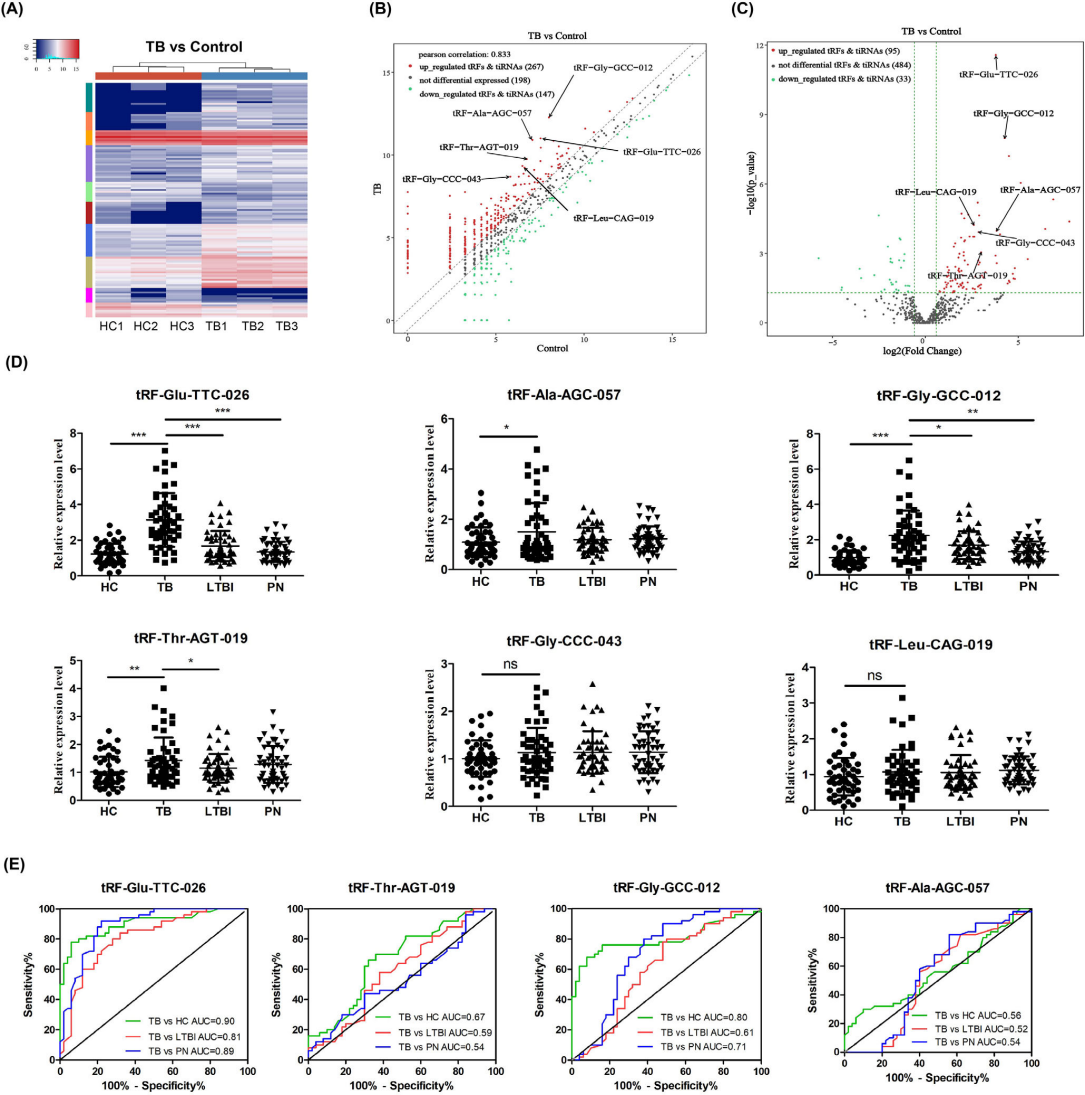
Identification of differentially expressed tsRNAs in PBMCs of TB patients. (A) A heat map showcasing the clustering of tsRNAs that display differential expression in three TB patients and three healthy individuals. (B) A comparison of tsRNAs between two groups through a scatter plot. (C) A differential analysis of tsRNAs displayed in a volcano plot. (D) Using qRT‐PCR, we measured the levels of tsRNAs in peripheral blood mononuclear cells (PBMCs) samples from 50 patients with active TB, 50 patients with pneumonia (PN), 50 with latent TB infection (LTBI) and 50 healthy controls (HC). (E) Utilising receiver operating characteristic (ROC) curve analysis to assess the diagnostic efficacy of validated tsRNAs. AUC, The area under the curve. ^*^ *p* < .05, ^**^ *p* < .01, ^***^ *p* < .001.

To identify the most suitable biomarkers for clinical practice, we selected six significantly up‐regulated tsRNAs for qRT‐PCR validation in the discovery cohort (Figure 2D, Table S2). Criteria for tsRNAs selection involved a lower *p*‐value, greater fold change, higher Counts Per Million (CPM) and a length of 18 bp or more. Compared with non‐TB (LTBI, PN and HC), TB patients exhibited markedly elevated levels of tRF‐Glu‐TTC‐026, tRF‐Ala‐AGC‐057, tRF‐Gly‐GCC‐012 and tRF‐Thr‐AGT‐019, the calculated The area under the curve (AUC) values range were from 0.52 to 0.90 (Figure 2E). In the combined analysis, a logistic regression model with tRF‐Gly‐GCC‐012 and tRF‐Glu‐TTC‐026 resulted in a slightly higher AUC of 0.88 compared with a single tRF‐Glu‐TTC‐026 (AUC = 0.87) and tRF‐Gly‐GCC‐012 (AUC = 0.71) in distinguishing TB from non‐TB (Figure S1). We found the potential use of tRF‐Glu‐TTC‐026 in distinguishing TB from LTBI, PN and HC (all AUC > 0.80). Amongst these tsRNAs, tRF‐Glu‐TTC‐026 was moderately correlated with lung injury severity (Figure S2). Based on the AUCs of the four confirmed tsRNAs in the discovery cohort and their correlations with the lung injury severity, we further evaluated the diagnostic value of tRF‐Glu‐TTC‐026 in TB.

We conducted tRF‐Glu‐TTC‐026 testing in an additional independent validation cohort. Consistent with the results above, tRF‐Glu‐TTC‐026 levels in TB patients increased significantly (Figure 3A). tRF‐Glu‐TTC‐026 achieved 79.44% sensitivity and 79.05% specificity in distinguishing TB patients from non‐TB individuals at a cut‐off point of 1.745 (Youden index = 0.584) (Figure 3B). Accuracy of tRF‐Glu‐TTC‐026 in distinguishing TB from LTBI, PN and HC was then further confirmed by using a separate “prediction” sample cohort (Figure 3C). tRF‐Glu‐TTC‐026 successfully differentiated TB from non‐TB, with 80.95% sensitivity, and 73.33% specificity (Figure 3D). After the anti‐TB treatment, the expression of tRF‐Glu‐TTC‐026 decreased (Figure 3E). After 3 months of treatment, the tRF‐Glu‐TTC‐026 level decreased from the initial average of 3.06 to 1.39, and then remained fairly stable (Figure 3F). These findings suggest that the tRF‐Glu‐TTC‐026 can be used for auxiliary diagnosis of TB.

**FIGURE 3:**
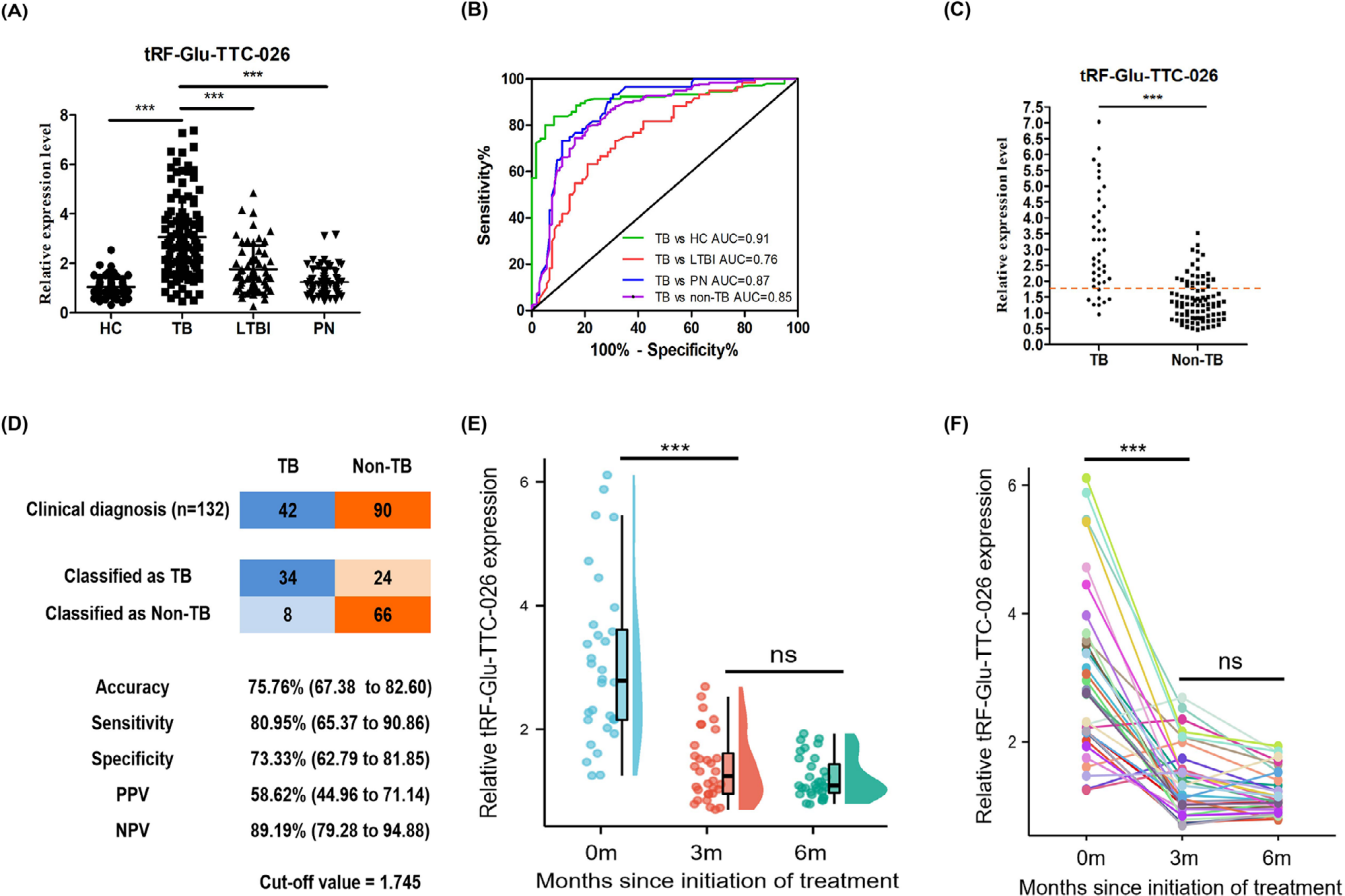
Expression of tRF‐Glu‐TTC‐026 in TB patients and its diagnostic value. (A) Representative plots showing tRF‐Glu‐TTC‐026 levels in PBMCs samples between HC (*n* = 60), TB (*n* = 105), LTBI (*n* = 60) and PN (*n* = 60). (B) Assessing the diagnostic accuracy of tRF‐Glu‐TTC‐026 using receiver operating characteristic (ROC) curve analysis. The non‐TB group includes LTBI, PN and HC. (C) A total of 132 participants (TB, *n* = 42; LTBI, *n* = 31; PN, *n* = 22; HC, *n* = 37) with clinical diagnostic results conducted independent validation using the established model, with a cutoff value set at 1.745 (dashed line). (D) List the numerical consistency and key parameters related to clinical diagnosis. (E,F) Before and after 3 and 6 months of the TB treatment, tRF‐Glu‐TTC‐026 expression was observed in patients who were microbiologically cured. AUC, The area under the curve; NPV, negative predictive value; PPV, positive predictive value. ^***^ *p* < .001; ns, no significance.

Our study then delved into the presence of tRF‐Glu‐TTC‐026 in monocyte/macrophages and its possible role in controlling the antibacterial capabilities against Mtb. Results indicated that Mtb H37Ra infection for 12 h caused an elevation in tRF‐Glu‐TTC‐026 levels in human monocytes, THP‐1 macrophages and monocyte‐derived macrophages (MDMs) (Figure 4A, Figure S3). Transfection of tRF‐Glu‐TTC‐026 mimic (Figure 4B) increases H37Ra's intracellular survival in MDMs, whereas tRF‐Glu‐TTC‐026 inhibitor decreases H37Ra's intracellular survival (Figure 4C).

**FIGURE 4:**
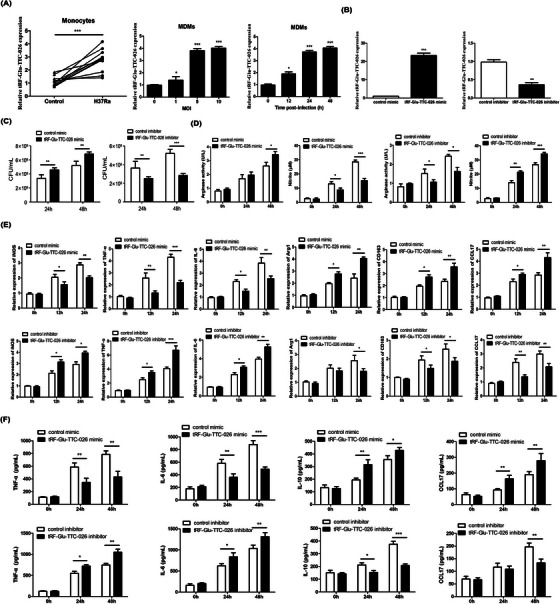
tRF‐Glu‐TTC‐026 promotes intracellular Mtb survival in macrophages by regulating macrophage polarization. (A) Following the sorting of human peripheral monocytes from PBMCs by immunomagnetic positive selection, the expression of tRF‐Glu‐TTC‐026 in the control group or H37Ra‐infected monocyte cells for 12 h was detected by qRT‐PCR. The expression of tRF‐Glu‐TTC‐026 in human monocyte‐derived macrophages (MDMs) infected with H37Ra for 12, 24 and 48 h was determined through qRT‐PCR analysis. MDMs were infected with H37Ra for 24 h at the specified multiplicity of infection (MOI) and qRT‐PCR results showed the presence of tRF‐Glu‐TTC‐026 expression. (B) The efficiencies of knockdown and overexpression of tRF‐Glu‐TTC‐026 were verified by qRT‐PCR. (C) MDMs pre‐transfected with tRF‐Glu‐TTC‐026 mimic, control mimic, tRF‐Glu‐TTC‐026 inhibitor, or control inhibitor were infected with H37Ra (MOI = 5) for a specified period of time, and then colony‐forming unit (CFU) detection was performed. (D) Analysis of nitrite production was conducted on the supernatants, whilst the lysates were tested for the arginase activity. (E) MDMs were transfected for 48 h with tRF‐Glu‐TTC‐026 mimic, control mimic, tRF‐Glu‐TTC‐026 inhibitor, or control inhibitor, and then for varying lengths of time, Mtb was added. The relative expression of different M1 markers (iNOS, IL‐6 and TNF‐α) and M2 markers (Arg1, CD163 and CCL17) was determined using qRT‐PCR.^9^ (F) The levels of cytokines released in the supernatants were measured at different time points using enzyme‐linked immunoabsorbent assay (ELISA). ^*^ *p* < .05, ^**^ *p* < .01, ^***^ *p* < .001.

Our previous research found that the polarization of macrophages significantly affects the intracellular survival of Mtb.^6^, ^7^ It has been demonstrated in recent studies that tsRNAs regulate macrophage polarization.^8^ The data revealed that silencing tRF‐Glu‐TTC‐026 expression led to a significant decrease in Mtb‐induced arginase activity and a notable increase in nitrite production (Figure 4D, Figure S3). During Mtb infection, the knockdown of tRF‐Glu‐TTC‐026 reduced the expressions M1 markers but increased the expression M2 markers, conversely, the overexpression of tRF‐Glu‐TTC‐026 had the opposite effect (Figure 4E). In addition, inhibiting tRF‐Glu‐TTC‐026 led to a reduction in interleukin‐10 (IL‐10) and CCL17 secretion, coupled with an increase in tumor necrosis factor (TNF‐α) and IL‐6) secretion (Figure 4F). Therefore, we believe that tRF‐Glu‐TTC‐026 can affect the intracellular survival of Mtb by inducing macrophage polarization towards the M2‐like phenotype.

tRF‐Glu‐TTC‐026 is a tRF‐3b generated from tRNA‐Glu‐TTC, with a length of 22 nucleotides (Figure S4). We found that tRF Glu‐TTC‐026 mainly presents in the cytoplasm and exhibits significant enrichment in Argonaute‐2 (AGO2) (Figure S5). By conducting Gene Ontology (GO) functional enrichment analysis, we identified that potential target genes are associated with functions adenylate cyclase‐activating dopamine receptor signalling pathway and glial cell development. The Kyoto Encyclopedia of Genes and Genomes (KEGG) biological pathway analysis demonstrated a notable enrichment in the regulation of the dopaminergic synapse and DNA replication (Figure S5).

This study revealed a potential unique elevation of tRF‐Glu‐TTC‐026 during active TB when compared to other types of PN. As is well known, hypoxia is an important stress encountered by Mtb in necrotic pulmonary granulomas and can lead to tsRNAs dysregulation. We speculate that the pattern of tsRNAs disorder induced by Mtb may be different from other intracellular bacterial pathogens with less toxicity. The tRF‐Glu‐TTC‐026 disorder may be species‐specific and related to Mtb infection and cell stress related to TB pathology. However, further research is needed to elucidate this mechanism. In addition, we found that tRF‐Glu‐TTC‐026 had a high negative predictive value (NPV) for diagnosing TB, which was higher than its positive predictive value (PPV), indicating that tRF‐Glu‐TTC‐026 has high screening reliability, meaning that this indicator can exclude true negative cases.

AUCs of TB diagnosed by RISK6 and RISK11 biomarkers reported by Scriba Laboratory were between 0.88–0.92.^10^ In this study, the combination of tRF‐Glu‐TTC‐026 and tRF‐Gly‐GCC‐012 performs comparably to RISK6 and RISK11 in diagnosing TB. This will be a rapid approach to diagnose TB and monitor treatment response using non‐sputum samples. Future research can further verify the diagnostic performance of tsRNAs and host blood transcriptomic biomarkers for TB head to head.

In summary, we revealed the expression profile of tsRNAs in PBMCs of TB patients for the first time and underscored the diagnostic capability of tRF‐Glu‐TTC‐026 for the disease. In addition, tRF‐Glu‐TTC‐026 has been proved to promote the survival of Mtb by regulating the polarization of macrophages, which can further explore and develop new tsRNA‐based TB treatment strategies for TB control.

## AUTHOR CONTRIBUTIONS

Zikun Huang, Junming Li and Aiping Le conceived and designed the study. Cuifen Xiong, Qing Luo, Yongqin Guo, Peng Fu, Haiyan Zhu and Yiping Peng contributed to the acquisition and analysis of the data. Jianqing Xu, Yang Guo and Cheng Qing conducted the statistical analysis. Zikun Huang wrote and edited this manuscript.

## FUNDING INFORMATION

The study was funded by National Natural Science Foundation of China, Grant numbers: 32060181, 82360322. Outstanding Youth Fund of Jiangxi Natural Science Foundation, Grant number: 20212ACB216006. Province Natural Science Foundation of Jiangxi Province, Grant number: 20232ACB206027. Project for High and Talent of Science and Technology Innovation in Jiangxi “double thousand plan”, Grant number: jxsq2019201094. Key R&D project of Jiangxi Province, Grant numbers: 20202BBG73026. Foundation ofNanchang Key Laboratory of Diagnosis of Infectious Diseases, Grant numbers: Hongkezi [2022] No. 233. Jiangxi Provincial Health Commission Science and Technology Planning Project, Grant numbers: 202210217.

## DATA AVA ILAB IL ITY STATEMENT

The dataset used in this study can be provided at the reasonable request of the corresponding author.

## ETHICS STATEMENT

This study has obtained ethical approval from the First Affiliated Hospital of Nanchang University [CDYFYYLK(11‐020)]. Written informed consent forms are obtained from all participants.

## Supporting information

Supporting information

## References

[1] World Health Organization . Global Tuberculosis Report 2023. WHO Press; 2023.

[2] Yates TA , Khan PY . Estimating annual risk of infection with Mycobacterium tuberculosis. Lancet Infect Dis. 2022;22(9):1276‐1277. 36029781 10.1016/S1473-3099(22)00454-6

[3] Liu B , Cao J , Wang X , Guo C , Liu Y , Wang T . Deciphering the tRNA‐derived small RNAs: origin, development, and future. Cell Death Dis. 2021;13(1):24. 34934044 10.1038/s41419-021-04472-3 PMC8692627

[4] Li X , Liu X , Zhao D , et al. tRNA‐derived small RNAs: novel regulators of cancer hallmarks and targets of clinical application. Cell Death Discov. 2021;7(1):249. 34537813 10.1038/s41420-021-00647-1 PMC8449783

[5] Yang K , Xiao Q , Wang K , et al. Circulating exosomal tsRNAs: potential biomarkers for large artery atherosclerotic stroke superior to plasma tsRNAs. Clin Transl Med. 2023;13(2):e1194. 36720657 10.1002/ctm2.1194 PMC9889266

[6] Huang Z , Luo Q , Guo Y , et al. Mycobacterium tuberculosis‐induced polarization of human macrophage orchestrates the formation and development of tuberculous granulomas in vitro. PLoS One. 2015;10(6):e0129744. 26091535 10.1371/journal.pone.0129744 PMC4474964

[7] Huang Z , Yao F , Liu J , et al. Up‐regulation of circRNA‐0003528 promotes mycobacterium tuberculosis associated macrophage polarization via down‐regulating miR‐224‐5p, miR‐324‐5p and miR‐488‐5p and up‐regulating CTLA4. Aging. 2020;12(24):25658‐25672. 33318319 10.18632/aging.104175 PMC7803570

[8] Lu S , Wei X , Tao L , et al. A novel tRNA‐derived fragment tRF‐3022b modulates cell apoptosis and M2 macrophage polarization via binding to cytokines in colorectal cancer. J Hematol Oncol. 2022;15(1):176. 36527118 10.1186/s13045-022-01388-z PMC9756499

[9] Viola A , Munari F , Sánchez‐Rodríguez R , Scolaro T , Castegna A . The metabolic signature of macrophage responses. Front Immunol. 2019;10:1462. 31333642 10.3389/fimmu.2019.01462 PMC6618143

[10] Mendelsohn SC , Fiore‐Gartland A , Penn‐Nicholson A , et al, CORTIS‐HR Study Team . Validation of a host blood transcriptomic biomarker for pulmonary tuberculosis in people living with HIV: a prospective diagnostic and prognostic accuracy study. Lancet Glob Health. 2021;9(6):e841‐e853. 33862012 10.1016/S2214-109X(21)00045-0 PMC8131200

