# Supplementary material for: tRF‐Glu‐TTC‐026 as novel diagnostic biomarkers for active tuberculosis and regulates intracellular survival of Mycobacterium tuberculosis in macrophages by regulating macrophage polarization
Source: Clin Transl Med. 2024 Jul 23;14(7):e1781. doi: 10.1002/ctm2.1781 (PMC11265989; doi:10.1002/ctm2.1781)
Supplement: Supplementary file 1 — Supporting information [file CTM2-14-e1781-s001.docx]

**Supplementary materials**

**Materials** **and** **methods**

**Participant characteristics and classification**

The study participants were recruited from the First Affiliated Hospital of Nanchang University and the Jiangxi Chest Hospital (Nanchang, China) between January 2021 and January 2023. In this study, participants were recruited into three cohorts (n = 678): the discovery cohort included 53 TB patients, 50 LTBI subjects, 50 PN subjects, and 53 healthy controls. In an independent validation cohort, we prospectively recruited 322 individuals; 37 cases were excluded, leaving 285 individuals for further analysis that included 105 TB patients, 60 LTBI subjects, 60 PN patients, and 60 healthy controls. In the prediction cohort, 150 individuals were recruited prospectively; 18 cases were excluded, leaving 132 individuals for further analysis that included 42 TB patients, 31 LTBI subjects, 22 PN patients, and 37 healthy controls.

The diagnosis of TB was established by examining the chest X-ray, positive sputum smears for AFB and/or Mtb positive cultures, and clinical symptoms.^19^ Although the IGRA (T-SPOT.TB) test was positive and the AFB smear and Mtb culture were negative, the LTBI subjects had a history of intimate contact with TB patients. PN cases include viral or non-Mtb bacterial infections of the upper or lower respiratory tract, negative Mtb cultures, negative AFB smears, and negative IGRA findings. Study subjects in the healthy control group did not have any close contact with TB patients in the past, had a normal chest X-ray, a negative IGRA, or any clinical indications of TB. Based on chest radiography, TB patients were then classified according to case severity, which included minimum, moderate, and advanced illness stages.^1,2^ Every X-ray examination was reviewed separately by two different clinical experts. In the event of divergent viewpoints, both sides will confer and determine the final score. Every participant had to be an adult (≥18 years old), and those who had a history of immunosuppressive drug use, diabetes, hypertension, autoimmune illnesses, or hepatitis B or hepatitis C infection were not included in the analysis.

**Blood collection and isolating human macrophages**

Exactly 5 mL of fresh venous blood were drawn into K2-EDTA tubes from study participants. According to the preceding description, fresh PBMCs were separated using density gradient centrifugation. Using immunomagnetic positive selection, monocytes were separated from PBMCs (STEMCELL Technologies, Vancouver, Canada). Monocytes were allowed to differentiate into human monocyte-derived macrophages (MDMs) for 1 week in complete media (RPMI 1640 + 4 mM L-glutamine + 10% fetal bovine serum).

**RNA isolation**

Following the manufacturer's instructions, Trizol reagent (Invitrogen, USA) was used to extract total RNA. Using a NanoDrop ND-1000 spectrophotometer (Agilent, Santa Clara, CA, USA), absorbance spectrometry was used to measure the absorbance ratios of A260/A280 and A 260/A230 in order to evaluate the concentration and quality of the RNA.

**Library preparation and tsRNA sequencing**

Library preparation comprised 0.3′-adapter ligation, 5′-adapter ligations, cDNA synthesis, and PCR amplification. We employed Agilent BioAnalyzer 2100 to quantify the sequencing libraries. The NextSeq 500/550 V2 kit (#FC-404-2005, Illumina) was used to perform the sequencing analysis following protocols from the manufacturer. The foregoing technique was conducted at Aksomics Biological Engineering Co., Ltd. (Shanghai, China) through the Illumina NextSeq 500 system (Illumina, CA, USA). Sequencing quality was examined in FastQC (Babraham Institute, Cambridge, UK). Further, the expression levels of both tRFs and tiRNA were measured and subsequently normalized to the number of transcripts per million of total aligned tRNA reads (TPM).

**Quantitative real-time PCR analysis**

The TRIzol reagent was used to isolate total RNA in accordance with the manufacturer's instructions. The quality and amount of RNA were assessed using a Thermo Fisher NanoDrop 2000 spectrophotometer. Hifair® Ⅱ 1st Strand cDNA Synthesis Kit (gDNA digester plus) for cDNA (Yeasen, Shanghai) or Mir-XTM miRNA First Strand synthesis kit (Takara) for RNA and tRFs, in accordance with the protocols, were used for reverse transcription. Using Hieff® qPCR SYBR Green Master Mix (High Rox Plus) (Yeasen, Shanghai) and the StepOne Plus real-time PCR equipment (Applied Biosystems, CA, USA), quantitative expression was carried out. **Table S1** lists the primer sequences. For mRNAs and tsRNAs, respectively, we employed GAPDH and U6 as internal references. The 2^-△△Ct^ technique was used to calculate the expression levels of the genes.

**THP-1 cells and Bacterial Culture**

The researchers procured THP-1 cells from the Shanghai Cell Bank in China (Type Culture Collection Committee of the Chinese Academy of Science). The cells were cultured at 37°C in an incubator with 5% CO_2_ in RPMI 1640 media (HyClone), which contained 10% fetal bovine serum (GIBCO). Following the addition of 100 nM phorbol 12-myristate 13-acetate (PMA, Sigma) to the well, the THP-1 cells were allowed to develop into macrophages and were kept there for three days. The American Type Culture Collection (ATCC) provided Mtb H37Ra. Middlebrook 7H9 broth supplemented with albumin, dextrose, and catalase was used to culture H37Ra.

**Cell transfection**

Using Lipofectamine 2000 (Invitrogen, USA) as directed by the manufacturer, macrophages were transiently transfected with 50 nM mimic negative control (mimic NC) or tRF-Glu-TTC-026 mimic (GenePharma, Shanghai, China); inhibitor negative control (inhibitor NC) or tRF-Glu-TTC-026 inhibitor (GenePharma, Shanghai, China) to investigate the possible function of tRF-Glu-TTC-026. Mimics and inhibitor sequence has been listed in Table S4.

**Cell infection**

H37Ra extracellular was eliminated by washing with PBS after a 4-hour Mtb H37Ra infection at a multiplicity of infection of 10. In RPMI 1640 medium supplemented with 10% FBS, THP-1 macrophages and MDMs were cultured for the specified durations.

**Colony-Forming Unit assay**

Colony-forming unit (CFU) assays were used to measure the viability of the bacteria inside human macrophages. tRF-Glu-TTC-026 mimic, tRF-Glu-TTC-026 inhibitor, mimic NC, or inhibitor NC were all transfected into THP-1 macrophages and MDMs for a duration of 24 hours. After infecting cells were lysed, homogenates were prepared on Middlebrook 7H10 agar plates supplemented with 10% OADC, and the mixture was incubated at 37°C for three to four weeks. Using conventional protocols, CFU was computed three times.

**Arginase activity assay**

The prior description of arginase activity assays was followed. Using 100 µl of 0.1% Triton X-100, cells were lysed by shaking them on a shaker at room temperature for 30 to 60 minutes. Next, a 100 µl solution of 25 mM Tris-HCl (pH 7.5) was introduced. 50 µl of 10 mM MnCl2 was added to 100 µl of this lysate, and the enzyme was heated for 10 to 15 minutes at 55°C to activate it. The lysate was incubated at 37°C for 15 to 120 minutes with 100 µl of 0.5M L-arginine (pH 9.7) in order to assess the hydrolysis of arginine. A 600 µl acid mixture containing 96% H2SO4, 85% H3PO4, and H2O at a ratio of 1:3:7 was added to terminate the process. The concentration of urea was measured at 540 nm after the addition of 40 µl of 9% α-isonitrosopropiophenone (ISPF) and heating at 100℃ for 45 to 60 min.

**Nitrite measurements**

Following the manufacturer's directions, centrifugation was used to remove supernatants after infection, and the Griess reaction was then used to test for NO. After the supernatant and standards were aliquoted into 96-well plates, the Griess reagent was added, and the mixture was left to sit at room temperature in the dark for ten minutes. Absorbance was therefore measured at 540 nm in an ELISA reader. The quantities of nitrite in supernatant were determined using a reference curve.

**Enzyme-linked immunoabsorbent assay (ELISA)**

For 24 hours, tRF-Glu-TTC-026 mimic, tRF-Glu-TTC-026 inhibitor, mimic NC, or inhibitor NC were transfected into THP-1 macrophages and MDMs. Following Mtb infection of THP-1 macrophages and MDMs, ELISA kits (R & D Systems, Minneapolis, MN, USA) were used to assess the quantities of TNF-α, IL-1β, IL-6, IL-10, and CCL17 in culture supernatants in accordance with the manufacturer's instructions. At 450 nm, the absorbance was measured with a microplate reader.

**Statistical analysis**

Statistical Package for Social Science (SPSS) version 26 (IBM, Chicago, USA) was used to perform all statistical analysis related to the present study. Inferential statistics such as one-way ANOVA test, paired *t*-test, Student *t*-test, or Mann-Whitney *U* test were conducted. The area under the curve (AUC) of receiver operating characteristic (ROC) curves was utilized to evaluate the sensitivity and specificity of the predictive ability of utilizing tsRNAs as TB biomarkers. The correlation study was conducted using the Spearman method. A *p*-value of less than 0.05 was deemed statistically significant.

**Reference**

1. Huang Z, Su R, Qing C, Peng Y, Luo Q, Li J. Plasma Circular RNAs hsa_circ_0001953 and hsa_circ_0009024 as Diagnostic Biomarkers for Active Tuberculosis. *Front Microbiol.* 2018; 9:2010.
2. Luo J, Zhang M, Yan B, Li F, Guan S, Chang K, Jiang W, Xu H, Yuan T, Chen M, Deng S. Diagnostic performance of plasma cytokine biosignature combination and MCP-1 as individual biomarkers for differentiating stages Mycobacterium tuberculosis infection. *J Infect.* 2019; 78(4):281-291.

**Supplemental Figures**


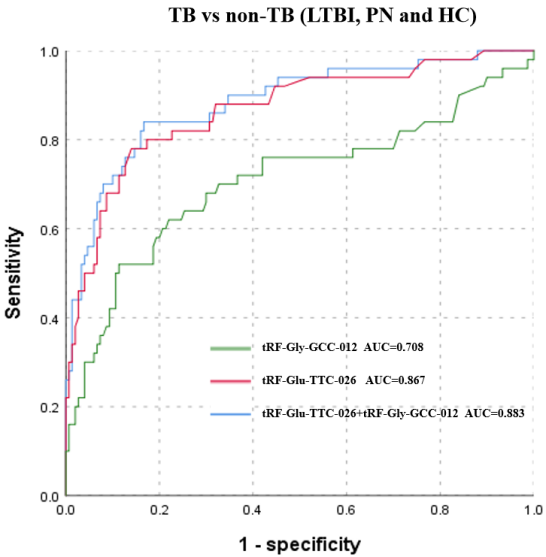


**Figure S1.** **Evaluation of combined diagnostic value of tRF-Gly-GCC-012 and tRF-Glu-TTC-026 in tuberculosis.** ROC curve combined diagnostic analysis of tRF-Gly-GCC-012 and tRF-Glu-TTC-026 in TB (n=50) and non-TB (PN patients, n=50; LTBI, n=50; HC, n=50). SPSS binary logistic regression was used to predict the probability of joint diagnosis. AUC, the area under the curve; ROC, receiver operating characteristic.

**
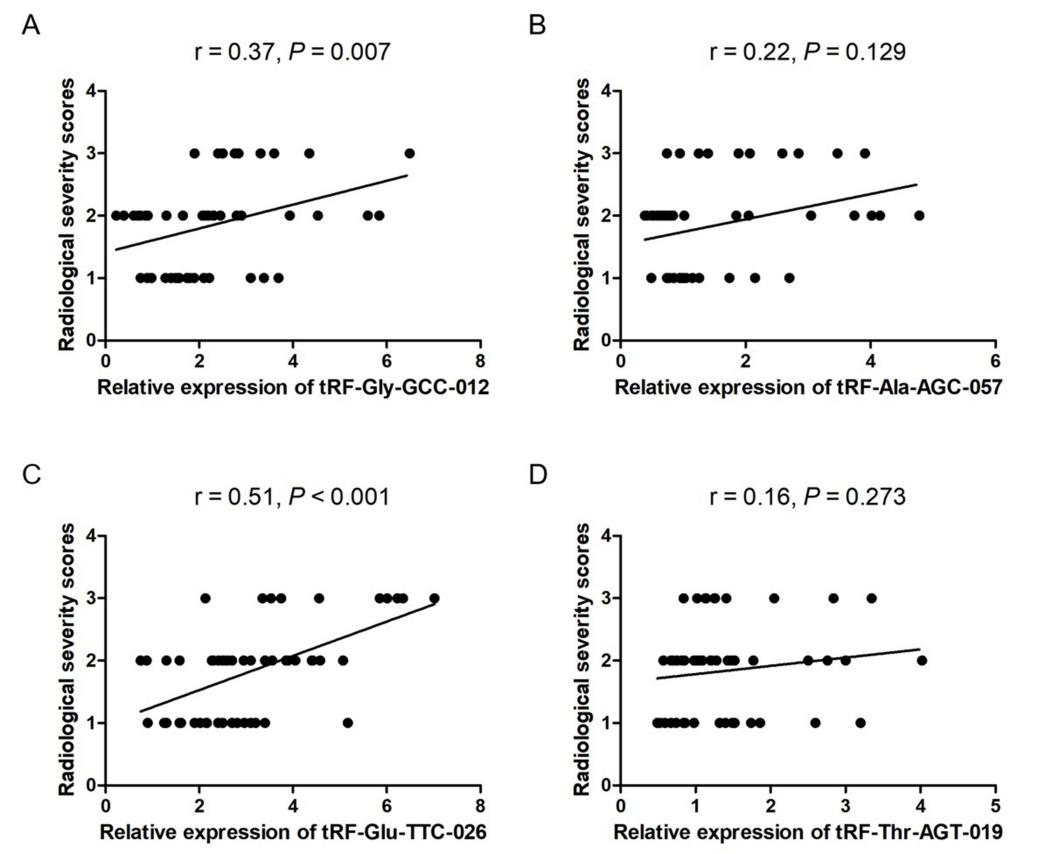
**

**Figure S2. Associations of validated tsRNAs levels with lung injury in tuberculosis patients.** In TB patients, lung damage was categorized into three classes using a double-blind process. The minimal (1) (n=18), moderate (2) (n=22), and advanced (3) (n=10) illness phases are represented by the images. By using the Spearman's rank correlation test, the amounts of verified tsRNAs were linked to the severity of lung damage in patients with active TB. Each chart specifies the values of *p* and *r*.

**
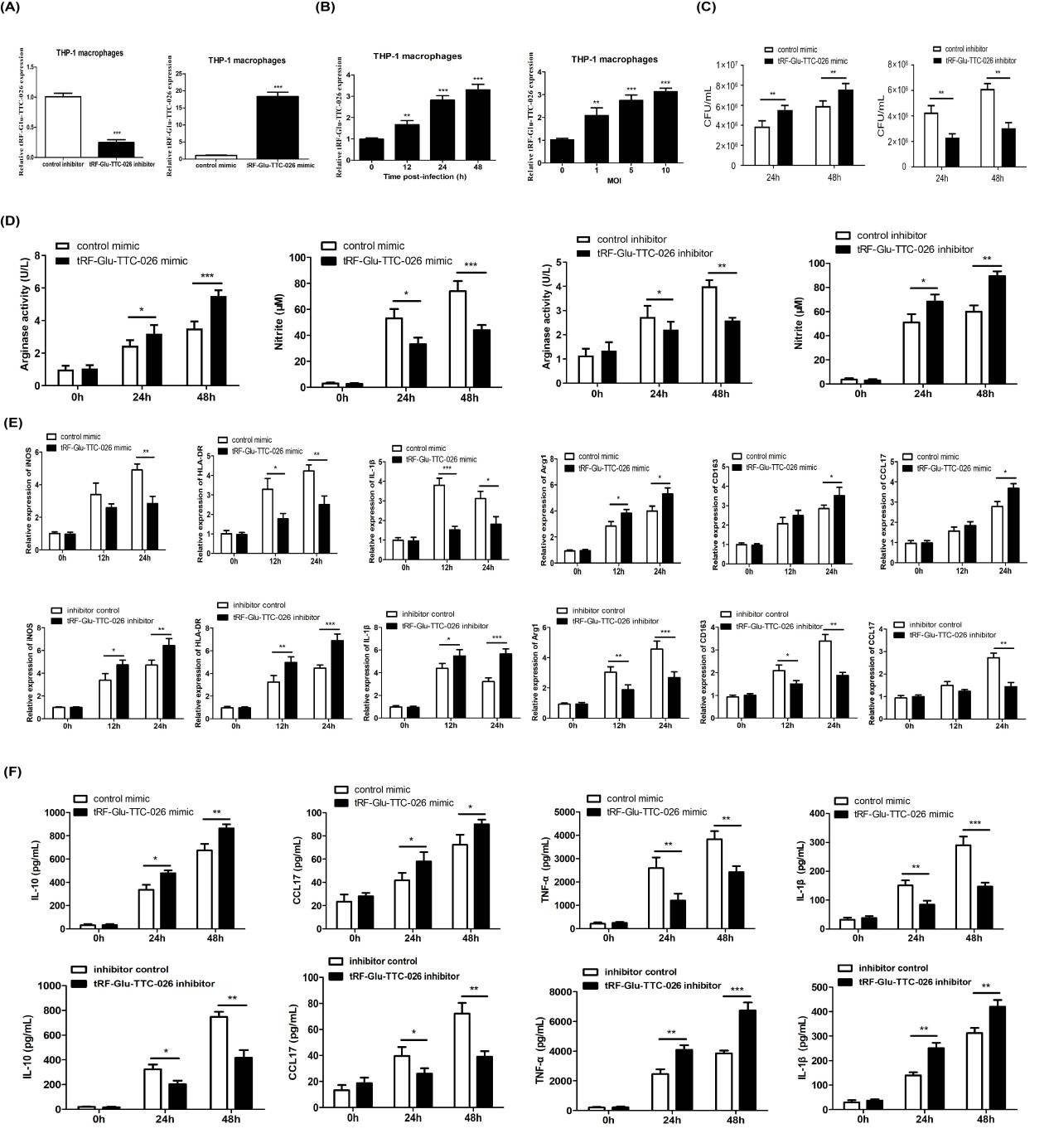
**

**Figure S3. tRF-Glu-TTC-026 promotes intracellular Mtb survival in THP-1 macrophages by regulating macrophage polarization.** (A) The efficiencies of knockdown and overexpression of tRF-Glu-TTC-026 were verified by qRT-PCR. (B) THP-1 macrophages were infected with H37Ra for 24 hours at the specified MOIs and qRT-PCR results showed the presence of tRF-Glu-TTC-026 expression. The expression of tRF-Glu-TTC-026 in THP-1 macrophages infected with H37Ra for 12, 24, and 48 hours was determined through qRT-PCR analysis. (C) THP-1 macrophages pre-transfected with tRF-Glu-TTC-026 mimic, control mimic, tRF-Glu-TTC-026 inhibitor, or control inhibitor were infected with H37Ra (MOI=5) for a specified period of time, and then CFU detection was performed. (D) Analysis of nitrite production was conducted on the supernatants, while the lysates were tested for arginase activity. (E) THP-1 macrophages were transfected for 48 hours with tRF-Glu-TTC-026 mimic, control mimic, tRF-Glu-TTC-026 inhibitor, or control inhibitor, and then for varying lengths of time, Mtb was added. The relative expression of different M1 markers (iNOS, HLA-DR, and IL-1β) and M2 markers (Arg1, CD163, and CCL17) was determined using qRT-PCR. (F) The levels of cytokines released in the supernatants were measured at different time points using ELISA. ^*^*p* < 0.05, ^**^*p* < 0.01, ^***^*p* < 0.001.

**
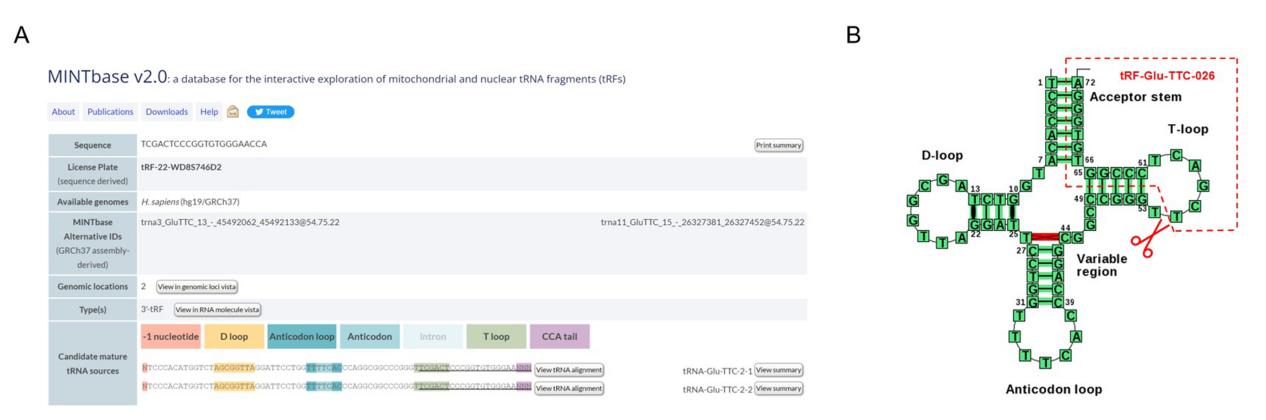
**

**Figure S4. Basic information of tRF-Glu-TTC-026.** (A) tRF-Glu-TTC-026 is a tRF-3b with a length of 22 nucleotides in the MINTbase v2.0. (B) tRF-Glu-TTC-026 is a degradation product of tRNA-Glu-TTC which specifically cleaves at the 3’ end of T-loop in tRNAdb.

**
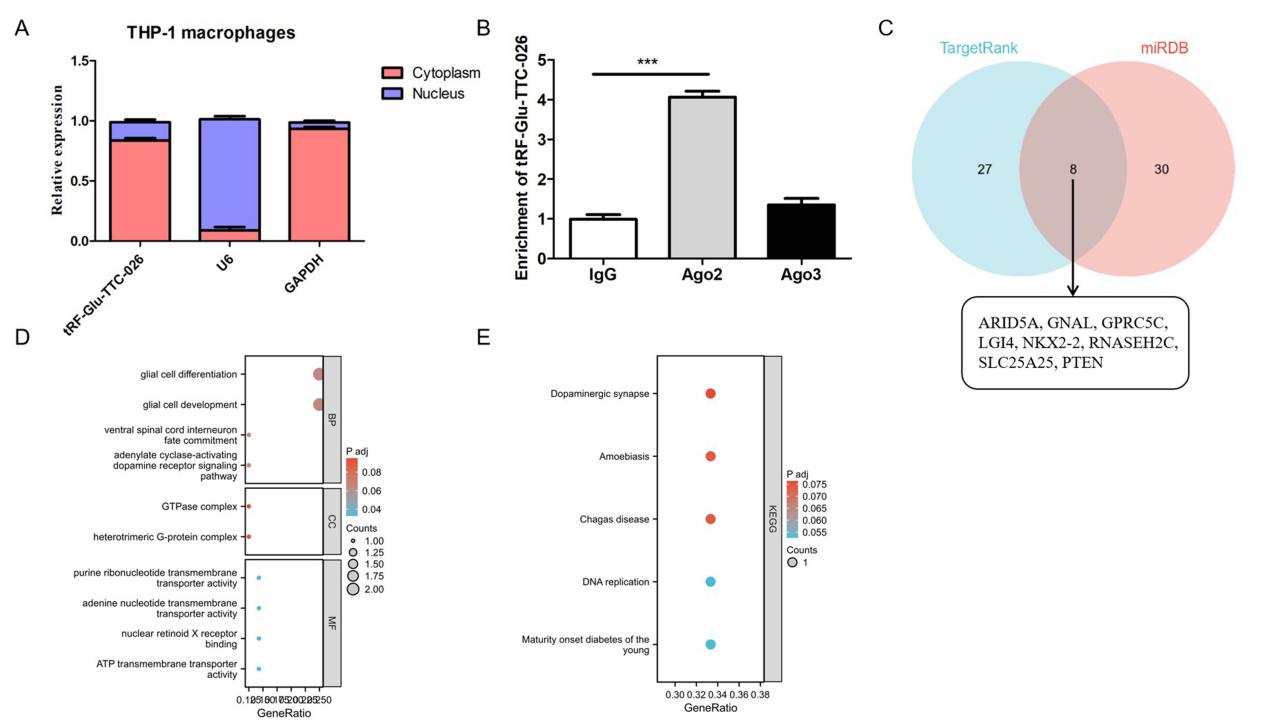
**

**Figure S5. Prediction of tRF-Glu-TTC-026 downstream in THP-1 macrophages.** (A) Nuclear and cytoplasmic RNA isolation assay was performed on THP-1 macrophages for the detection of tRF-Glu-TTC-026. (B) The enrichment between AGO2, AGO3, and tRF-Glu-TTC-026 was detected by RIP-qPCR. (C) Venn diagram evaluated the overlapped genes among TargetRank and miRDB predictions. (D) GO analysis of the tRF-Glu-TTC-026 target genes enriched in biological process (BP), cellular component (CC), and molecular function (MF). (E) KEGG pathway analysis of tRF-Glu-TTC-026.

**Supplemental Tables**

| **Table S1.** Demographic characteristics in this study | | | | | | | | | | | | |
| --- | --- | --- | --- | --- | --- | --- | --- | --- | --- | --- | --- | --- |
| Cohort | I | | | | II | | | | III | | | |
| Study part | Biomarker screening | | | | Biomarker validation | | | | Prediction | | | |
| Group | TB | LTBI | PN | HC | TB | LTBI | PN | HC | TB | LTBI | PN | HC |
| Cases (n) | 53 | 50 | 50 | 53 | 105 | 60 | 60 | 60 | 42 | 31 | 22 | 37 |
| Gender: male, n (%) | 29 (54.7) | 26 (52) | 28 (56) | 28 (52.8) | 62 (59) | 34 (56.7) | 38 (63.3) | 35 (58.3) | 20 (47.6) | 17 (54.8) | 10 (45.5) | 20 (54) |
| Age: median (IQR) | 54 (33-65) | 51 (31-60) | 58 (42-68) | 48 (30-58) | 53 (34-61) | 49 (28-63) | 56 (45-69) | 45 (32-59) | 49 (30-61) | 38 (27-58) | 57 (43-65) | 40 (28-50) |
| MONO% (%) | 7.6 (6.0–10.2) | 7.0 (5.9–9.0) | 6.4 (5.2-8.3) | 6.6 (4.9-7.9) | 7.5 (6.1–10.3) | 7.0 (5.6–8.9) | 6.5 (5.0-7.8) | 6.6 (5.2-8.3) | 7.3 (5.4–9.6) | 6.9 (5.2–9.5) | 6.5 (5.0-8.7) | 6.3 (4.6-8.5) |
| MONO# (×10^9^/L) | 0.52 (0.36–0.68) | 0.43 (0.31–0.53) | 0.38 (0.28–0.47) | 0.41 (0.30–0.52) | 0.50 (0.32–0.65) | 0.44 (0.32–0.56) | 0.39 (0.30–0.49) | 0.42 (0.31–0.51) | 0.50 (0.35–0.67) | 0.42 (0.32–0.54) | 0.39 (0.30–0.49) | 0.41 (0.32–0.50) |
| HIV status: positive, n (%) | 0 (0) | 0 (0) | 0 (0) | 0 (0) | 0 (0) | 0 (0) | 0 (0) | 0 (0) | 0 (0) | 0 (0) | 0 (0) | 0 (0) |
| IGRAs: positive, n (%) | 52 (98.1) | 50 (100) | 0 (0) | 0 (0) | 102 (97.1) | 60 (100) | 0 (0) | 0 (0) | 42 (100) | 31 (100) | 0 (0) | 0 (0) |
| History of TB: yes, n (%) | 5 (9.4) | 0 (0) | 0 (0) | 0 (0) | 12 (11.4) | 0 (0) | 2 (3.3) | 0 (0) | 3 (7.1) | 0 (0) | 0 (0) | 0 (0) |
| Any TB symptoms: yes, n (%) | 53 (100) | 0 (0) | 39 (78) | 0 (0) | 105 (100) | 0 (0) | 44 (73.3) | 0 (0) | 42 (100) | 0 (0) | 15 (68.2) | 0 (0) |
| Sputum AFB smears: positive, n (%) | 14 (26.4) | NA | 0 (0) | NA | 30 (28.6) | NA | 0 (0) | NA | 13 (31) | NA | 0 (0) | NA |
| Sputum Mtb cultures: positive, n (%) | 48 (90.6) | NA | 0 (0) | NA | 93 (88.6) | NA | 0 | NA | 39 (92.9) | NA | 0 (0) | NA |
| Status of chest radiograph |  |  |  |  |  |  |  |  |  |  |  |  |
| Minimal, n (%) | 19 (35.8) | NA | NA | NA | 43 (41) | NA | NA | NA | 13 (31) | NA | NA | NA |
| Moderate, n (%) | 23 (43.4) | NA | NA | NA | 44 (41.9) | NA | NA | NA | 19 (45.2) | NA | NA | NA |
| Advanced, n (%) | 11 (20.8) | NA | NA | NA | 18 (17.1) | NA | NA | NA | 10 (23.8) | NA | NA | NA |

TB, tuberculosis; Mtb, *Mycobacterium tuberculosis*; LTBI, latent TB infection; PN, pneumonia; HC, healthy control; AFB, acid-fast bacilli; IGRAs, interferon gamma release assays; NA, not applicable; IQR, interquartile ranges; MONO, monocytes.

**Table S2.** Difference of tsRNAs expression profile in peripheral blood mononuclear cells between TB patients and healthy subjects

| **Annotation** |  |  | **CPM** | | | | | |
| --- | --- | --- | --- | --- | --- | --- | --- | --- |
| **tsRNA_ID** | **Fold_Change** | **p_value** | **TB1** | **TB2** | **TB3** | **HC1** | **HC2** | **HC3** |
| tRF-Gly-GCC-037 | 215.8156 | 0.0000 | 497.55 | 64.40 | 69.65 | 0.00 | 0.00 | 0.00 |
| tRF-Tyr-GTA-039 | 118.3341 | 0.0000 | 212.59 | 64.40 | 69.65 | 0.00 | 0.00 | 0.00 |
| tRF-Glu-TTC-071 | 87.6859 | 0.0001 | 167.36 | 48.30 | 37.99 | 0.00 | 0.00 | 0.00 |
| tRF-Asn-GTT-033 | 45.6927 | 0.0018 | 36.19 | 40.25 | 63.32 | 0.00 | 0.00 | 0.00 |
| tRF-Tyr-GTA-040 | 35.1822 | 0.0000 | 361.86 | 96.60 | 82.32 | 0.00 | 0.00 | 12.04 |
| tRF-Thr-AGT-018 | 28.2917 | 0.0125 | 54.28 | 24.15 | 0.00 | 0.00 | 0.00 | 0.00 |
| tRF-Asp-GTC-009 | 28.2225 | 0.0044 | 27.14 | 32.20 | 25.33 | 0.00 | 0.00 | 0.00 |
| tRF-Gly-TCC-007 | 26.9736 | 0.0121 | 9.05 | 64.40 | 12.66 | 0.00 | 0.00 | 0.00 |
| tRF-Glu-CTC-014 | 26.2137 | 0.0077 | 22.62 | 24.15 | 31.66 | 0.00 | 0.00 | 0.00 |
| tRF-Ser-TGA-020 | 23.2282 | 0.0173 | 13.57 | 40.25 | 19.00 | 0.00 | 0.00 | 0.00 |
| tRF-Asp-GTC-025 | 22.7045 | 0.0150 | 18.09 | 32.20 | 19.00 | 0.00 | 0.00 | 0.00 |
| tRF-Glu-TTC-007 | 22.6477 | 0.0000 | 158.31 | 160.99 | 354.61 | 0.00 | 12.23 | 12.04 |
| tRF-Gln-TTG-042 | 21.5439 | 0.0198 | 9.05 | 40.25 | 19.00 | 0.00 | 0.00 | 0.00 |
| tRF-Gly-GCC-012 | 19.3153 | 0.0000 | 9887.73 | 3058.84 | 1925.00 | 238.27 | 146.82 | 373.34 |
| tRF-Asn-GTT-001 | 16.2505 | 0.0001 | 63.32 | 48.30 | 151.97 | 0.00 | 0.00 | 12.04 |
| tRF-Asp-GTC-044 | 14.4030 | 0.0026 | 167.36 | 24.15 | 25.33 | 0.00 | 12.23 | 0.00 |
| tRF-Ala-AGC-057 | 14.2898 | 0.0001 | 4468.93 | 668.12 | 557.24 | 105.90 | 73.41 | 216.78 |
| tRF-Glu-TTC-026 | 13.8781 | 0.0000 | 3197.91 | 1191.34 | 2013.65 | 119.14 | 207.99 | 108.39 |
| tRF-Gln-CTG-003 | 13.7205 | 0.0012 | 126.65 | 56.35 | 25.33 | 0.00 | 0.00 | 12.04 |
| tRF-Gln-TTG-003 | 9.3792 | 0.0064 | 36.19 | 80.50 | 37.99 | 0.00 | 12.23 | 0.00 |
| tRF-Asp-GTC-004 | 8.7449 | 0.0108 | 18.09 | 72.45 | 56.99 | 13.24 | 0.00 | 0.00 |
| tRF-Trp-CCA-006 | 8.2909 | 0.0091 | 40.71 | 48.30 | 44.33 | 13.24 | 0.00 | 0.00 |
| tRF-Thr-AGT-019 | 8.0367 | 0.0009 | 1981.17 | 370.28 | 196.30 | 79.42 | 122.35 | 108.39 |
| tRF-Ser-TGA-011 | 8.0067 | 0.0224 | 31.66 | 8.05 | 88.65 | 0.00 | 12.23 | 0.00 |
| tRF-Gln-TTG-041 | 7.8396 | 0.0317 | 18.09 | 16.10 | 94.98 | 13.24 | 0.00 | 0.00 |
| tRF-Thr-TGT-015 | 7.6028 | 0.0025 | 54.28 | 88.55 | 82.32 | 0.00 | 12.23 | 12.04 |
| tRF-Ala-AGC-063 | 7.5032 | 0.0241 | 67.85 | 24.15 | 19.00 | 0.00 | 0.00 | 12.04 |
| tRF-Gly-TCC-055 | 7.4620 | 0.0000 | 348.29 | 1376.48 | 2729.20 | 158.85 | 256.93 | 120.43 |
| tRF-Gly-CCC-043 | 7.3152 | 0.0001 | 863.93 | 233.44 | 120.31 | 52.95 | 48.94 | 60.22 |
| tRF-Val-TAC-015 | 7.2781 | 0.0031 | 63.32 | 88.55 | 63.32 | 26.47 | 0.00 | 0.00 |
| tRF-Ser-GCT-032 | 7.2299 | 0.0020 | 31.66 | 104.64 | 278.62 | 0.00 | 24.47 | 24.09 |
| tRF-Ile-AAT-003 | 7.1566 | 0.0270 | 18.09 | 64.40 | 37.99 | 13.24 | 0.00 | 0.00 |
| tRF-Gly-TCC-056 | 7.0829 | 0.0000 | 199.02 | 660.07 | 1196.79 | 66.19 | 122.35 | 72.26 |
| tRF-Asn-GTT-042 | 6.5066 | 0.0463 | 13.57 | 24.15 | 69.65 | 13.24 | 0.00 | 0.00 |
| tRF-Arg-TCG-002 | 6.4565 | 0.0099 | 31.66 | 88.55 | 75.99 | 26.47 | 0.00 | 0.00 |
| tRF-Lys-CTT-037 | 6.3355 | 0.0345 | 22.62 | 56.35 | 25.33 | 0.00 | 0.00 | 12.04 |
| tRF-Glu-CTC-001 | 6.2232 | 0.0002 | 122.13 | 120.74 | 272.29 | 26.47 | 12.23 | 36.13 |
| tRF-Leu-CAG-019 | 6.0945 | 0.0001 | 1162.46 | 386.38 | 234.29 | 92.66 | 61.17 | 132.48 |
| tRF-Ala-CGC-008 | 5.9684 | 0.0002 | 3785.93 | 861.31 | 854.85 | 251.51 | 244.69 | 409.47 |
| tRF-Val-CAC-047 | 5.8755 | 0.0320 | 126.65 | 24.15 | 6.33 | 13.24 | 0.00 | 12.04 |
| tRF-Leu-CAA-017 | 5.2215 | 0.0002 | 746.33 | 297.83 | 177.30 | 79.42 | 97.88 | 48.17 |
| tRF-Glu-CTC-003 | 4.9651 | 0.0000 | 325.67 | 458.83 | 690.21 | 52.95 | 122.35 | 96.35 |
| tRF-Lys-CTT-030 | 4.8956 | 0.0031 | 131.17 | 112.69 | 88.65 | 26.47 | 24.47 | 12.04 |
| tRF-Gln-TTG-012 | 4.8587 | 0.0177 | 22.62 | 88.55 | 101.32 | 13.24 | 0.00 | 24.09 |
| tRF-Ser-AGA-018 | 4.6478 | 0.0342 | 126.65 | 24.15 | 31.66 | 0.00 | 24.47 | 12.04 |
| tRF-Lys-CTT-006 | 4.3933 | 0.0003 | 348.29 | 249.54 | 265.95 | 52.95 | 36.70 | 96.35 |
| tRF-Leu-AAG-004 | 4.3596 | 0.0020 | 194.50 | 209.29 | 126.64 | 79.42 | 12.23 | 24.09 |
| tRF-Tyr-GTA-003 | 4.2896 | 0.0003 | 389.00 | 281.74 | 234.29 | 66.19 | 73.41 | 60.22 |
| tRF-Lys-TTT-010 | 4.2787 | 0.0000 | 710.14 | 499.07 | 1253.78 | 185.32 | 146.82 | 204.74 |
| tRF-Gln-CTG-027 | 4.2015 | 0.0009 | 470.41 | 257.59 | 139.31 | 66.19 | 48.94 | 84.30 |
| tRF-Gly-TCC-057 | 4.1744 | 0.0279 | 31.66 | 80.50 | 126.64 | 0.00 | 36.70 | 12.04 |
| tRF-Gly-CCC-009 | 4.1740 | 0.0012 | 221.64 | 249.54 | 145.64 | 39.71 | 73.41 | 24.09 |
| tRF-Gly-GCC-001 | 4.0227 | 0.0089 | 81.42 | 104.64 | 88.65 | 0.00 | 24.47 | 36.13 |
| tRF-Tyr-GTA-013 | 4.0214 | 0.0309 | 45.23 | 56.35 | 69.65 | 26.47 | 0.00 | 12.04 |
| tRF-Trp-CCA-010 | 4.0040 | 0.0183 | 149.27 | 40.25 | 75.99 | 26.47 | 36.70 | 0.00 |
| tRF-Ala-AGC-064 | 3.9808 | 0.0200 | 257.82 | 72.45 | 31.66 | 52.95 | 24.47 | 12.04 |
| tRF-Lys-CTT-008 | 3.8613 | 0.0000 | 1782.14 | 1287.93 | 1431.09 | 304.46 | 256.93 | 553.99 |
| tRF-Val-AAC-042 | 3.8460 | 0.0056 | 244.25 | 112.69 | 88.65 | 26.47 | 24.47 | 60.22 |
| tRF-Thr-TGT-016 | 3.8158 | 0.0245 | 67.85 | 48.30 | 94.98 | 0.00 | 48.94 | 0.00 |
| tRF-Lys-CTT-007 | 3.8123 | 0.0002 | 547.31 | 603.72 | 544.57 | 52.95 | 159.05 | 204.74 |
| tRF-Val-AAC-006 | 3.7400 | 0.0234 | 72.37 | 56.35 | 75.99 | 26.47 | 12.23 | 12.04 |
| tiRNA-Gly-CCC-003 | 3.6968 | 0.0219 | 67.85 | 56.35 | 132.98 | 52.95 | 0.00 | 12.04 |
| tRF-Lys-CTT-018 | 3.5813 | 0.0010 | 217.11 | 378.33 | 728.21 | 105.90 | 134.58 | 96.35 |
| tRF-Ser-GCT-108 | 3.5695 | 0.0061 | 90.46 | 185.14 | 265.95 | 26.47 | 73.41 | 36.13 |
| tRF-Pro-AGG-024 | 3.4983 | 0.0045 | 253.30 | 193.19 | 107.65 | 66.19 | 48.94 | 36.13 |
| tRF-Pro-TGG-021 | 3.4930 | 0.0271 | 108.56 | 241.49 | 25.33 | 26.47 | 24.47 | 48.17 |
| tRF-Ser-GCT-003 | 3.4439 | 0.0015 | 325.67 | 305.88 | 272.29 | 92.66 | 24.47 | 132.48 |
| tRF-Ser-GCT-109 | 3.4314 | 0.0053 | 140.22 | 209.29 | 164.64 | 39.71 | 73.41 | 24.09 |
| tRF-Tyr-GTA-014 | 3.2985 | 0.0200 | 76.89 | 96.60 | 145.64 | 66.19 | 0.00 | 24.09 |
| tRF-Lys-TTT-002 | 3.0974 | 0.0133 | 194.50 | 104.64 | 145.64 | 52.95 | 12.23 | 72.26 |
| tRF-Ser-GCT-008 | 3.0748 | 0.0126 | 99.51 | 144.89 | 259.62 | 26.47 | 73.41 | 48.17 |
| tRF-Gly-TCC-054 | 3.0705 | 0.0038 | 443.27 | 225.39 | 246.96 | 66.19 | 110.11 | 108.39 |
| tiRNA-Lys-CTT-003 | 3.0431 | 0.0004 | 1194.13 | 837.16 | 1304.44 | 277.99 | 391.51 | 361.30 |
| tRF-Arg-ACG-014 | 2.9788 | 0.0417 | 54.28 | 104.64 | 88.65 | 26.47 | 12.23 | 36.13 |
| tRF-Val-CAC-017 | 2.9171 | 0.0467 | 27.14 | 225.39 | 113.98 | 39.71 | 24.47 | 48.17 |
| tRF-Val-AAC-016 | 2.8970 | 0.0005 | 2569.18 | 4588.26 | 2418.92 | 701.59 | 880.90 | 1565.62 |
| tRF-Asp-GTC-019 | 2.8677 | 0.0420 | 94.99 | 40.25 | 170.97 | 13.24 | 36.70 | 48.17 |
| tRF-Tyr-GTA-015 | 2.7866 | 0.0488 | 104.03 | 48.30 | 107.65 | 26.47 | 48.94 | 12.04 |
| tiRNA-Val-AAC-002 | 2.7619 | 0.0145 | 126.65 | 209.29 | 227.96 | 79.42 | 48.94 | 60.22 |
| tRF-Val-AAC-015 | 2.6038 | 0.0262 | 167.36 | 249.54 | 132.98 | 13.24 | 85.64 | 96.35 |
| tRF-Gly-CCC-007 | 2.5576 | 0.0155 | 370.90 | 217.34 | 348.27 | 39.71 | 171.29 | 132.48 |
| tRF-Pro-TGG-029 | 2.4119 | 0.0052 | 981.54 | 1054.50 | 2159.29 | 397.12 | 611.73 | 602.16 |
| tRF-Tyr-GTA-002 | 2.3916 | 0.0090 | 1252.93 | 660.07 | 728.21 | 304.46 | 244.69 | 517.86 |
| tRF-Ser-GCT-107 | 2.3556 | 0.0066 | 836.79 | 1078.64 | 1070.15 | 291.22 | 550.56 | 337.21 |
| tRF-Val-AAC-004 | 2.3370 | 0.0191 | 547.31 | 313.93 | 303.95 | 185.32 | 171.29 | 120.43 |
| tiRNA-Gly-CCC-002 | 2.3265 | 0.0066 | 841.32 | 885.45 | 1431.09 | 383.89 | 513.86 | 361.30 |
| tRF-Val-AAC-005 | 2.2831 | 0.0039 | 9507.78 | 4056.99 | 6015.63 | 2409.22 | 2630.45 | 3179.42 |
| tRF-Lys-CTT-002 | 2.2623 | 0.0100 | 1067.48 | 1062.55 | 911.84 | 317.70 | 293.63 | 674.42 |
| tRF-Gly-CCC-031 | 2.2272 | 0.0228 | 361.86 | 273.69 | 367.27 | 145.61 | 146.82 | 132.48 |
| tRF-Ala-AGC-060 | 2.1941 | 0.0284 | 958.92 | 450.78 | 493.91 | 357.41 | 146.82 | 337.21 |
| tRF-Pro-TGG-010 | 2.1209 | 0.0174 | 1162.46 | 869.36 | 1374.10 | 344.17 | 709.61 | 445.60 |
| tRF-Gly-CCC-006 | 2.0296 | 0.0329 | 502.08 | 442.73 | 481.25 | 277.99 | 171.29 | 216.78 |
| tRF-Val-TAC-020 | 2.0079 | 0.0465 | 497.55 | 297.83 | 329.28 | 198.56 | 134.58 | 204.74 |
| tRF-Glu-CTC-008 | 1.7502 | 0.0456 | 2324.93 | 3485.47 | 2361.93 | 1217.85 | 1284.64 | 1926.92 |
| tRF-Val-AAC-003 | 1.7016 | 0.0422 | 12456.92 | 6858.25 | 9707.32 | 5268.52 | 5016.21 | 5961.41 |
| tRF-Phe-GAA-007 | 0.0187 | 0.0016 | 0.00 | 0.00 | 0.00 | 52.95 | 97.88 | 0.00 |
| tRF-Trp-TCA-002 | 0.0439 | 0.0375 | 0.00 | 0.00 | 0.00 | 39.71 | 24.47 | 0.00 |
| tRF-Cys-GCA-008 | 0.0446 | 0.0296 | 0.00 | 0.00 | 0.00 | 13.24 | 36.70 | 12.04 |
| tRF-Phe-GAA-030 | 0.0861 | 0.0047 | 0.00 | 8.05 | 6.33 | 105.90 | 61.17 | 0.00 |
| tRF-Glu-TTC-033 | 0.0912 | 0.0115 | 4.52 | 0.00 | 0.00 | 39.71 | 48.94 | 0.00 |
| tRF-Glu-TTC-072 | 0.1497 | 0.0087 | 4.52 | 0.00 | 19.00 | 105.90 | 48.94 | 12.04 |
| tRF-Arg-ACG-017 | 0.1764 | 0.0000 | 212.59 | 112.69 | 88.65 | 1456.12 | 403.74 | 433.56 |
| tRF-Leu-AAG-009 | 0.1937 | 0.0435 | 4.52 | 8.05 | 6.33 | 52.95 | 24.47 | 24.09 |
| tRF-Pro-TGG-003 | 0.2194 | 0.0363 | 9.05 | 8.05 | 12.66 | 39.71 | 85.64 | 12.04 |
| tRF-Leu-TAG-028 | 0.2476 | 0.0005 | 54.28 | 32.20 | 69.65 | 277.99 | 122.35 | 216.78 |
| tRF-Glu-TTC-027 | 0.2651 | 0.0135 | 40.71 | 482.98 | 576.23 | 1217.85 | 1651.68 | 879.16 |
| tRF-Ile-AAT-020 | 0.2737 | 0.0023 | 22.62 | 64.40 | 44.33 | 145.61 | 159.05 | 132.48 |
| tRF-Leu-AAG-008 | 0.2747 | 0.0017 | 58.80 | 64.40 | 31.66 | 251.51 | 171.29 | 120.43 |
| tRF-Gly-TCC-010 | 0.2799 | 0.0181 | 18.09 | 48.30 | 19.00 | 52.95 | 61.17 | 168.61 |
| tiRNA-His-GTG-002 | 0.2884 | 0.0224 | 18.09 | 48.30 | 25.33 | 132.37 | 146.82 | 12.04 |
| tRF-Glu-TTC-018 | 0.2905 | 0.0025 | 54.28 | 136.84 | 69.65 | 423.60 | 146.82 | 264.95 |
| tRF-Lys-TTT-027 | 0.2980 | 0.0218 | 4.52 | 56.35 | 31.66 | 52.95 | 110.11 | 108.39 |
| tRF-Gln-CTG-030 | 0.3154 | 0.0453 | 18.09 | 16.10 | 12.66 | 39.71 | 48.94 | 60.22 |
| tRF-SeC-TCA-008 | 0.3369 | 0.0169 | 40.71 | 64.40 | 19.00 | 105.90 | 110.11 | 132.48 |
| tRF-Val-CAC-005 | 0.3387 | 0.0362 | 36.19 | 32.20 | 12.66 | 105.90 | 48.94 | 84.30 |
| tRF-Val-TAC-005 | 0.3684 | 0.0396 | 49.76 | 112.69 | 37.99 | 317.70 | 61.17 | 132.48 |
| tRF-His-GTG-008 | 0.3771 | 0.0020 | 1044.86 | 829.11 | 531.91 | 3018.15 | 1480.39 | 1613.80 |
| tRF-Gln-TTG-013 | 0.3836 | 0.0002 | 1899.75 | 2334.38 | 2444.24 | 5599.46 | 4844.93 | 5949.37 |
| tRF-Gln-CTG-005 | 0.3976 | 0.0003 | 6033.96 | 5047.09 | 5053.13 | 14428.87 | 10668.62 | 13476.41 |
| tRF-Leu-TAG-015 | 0.4056 | 0.0042 | 357.33 | 515.17 | 519.24 | 1509.07 | 636.20 | 1083.89 |
| tRF-Leu-AAG-007 | 0.4512 | 0.0021 | 5346.43 | 4056.99 | 5034.13 | 12655.04 | 8368.51 | 9249.22 |
| tRF-Leu-AAG-001 | 0.4597 | 0.0011 | 27234.24 | 28141.35 | 35397.22 | 70873.54 | 56499.66 | 57867.86 |
| tRF-Val-AAC-020 | 0.4787 | 0.0247 | 176.41 | 193.19 | 297.62 | 423.60 | 513.86 | 361.30 |
| tRF-iMet-CAT-020 | 0.5216 | 0.0447 | 235.21 | 321.98 | 265.95 | 648.64 | 391.51 | 445.60 |
| tRF-Leu-CAA-006 | 0.5224 | 0.0247 | 782.52 | 740.56 | 721.88 | 1217.85 | 1627.21 | 1204.33 |
| tRF-Glu-TTC-009 | 0.5267 | 0.0428 | 339.24 | 708.36 | 525.58 | 1284.04 | 709.61 | 794.86 |
| tRF-Val-AAC-001 | 0.5534 | 0.0255 | 4061.84 | 4749.26 | 5015.13 | 10682.66 | 6900.35 | 5804.85 |
| tRF-Ser-TGA-006 | 0.5986 | 0.0467 | 18318.99 | 16461.40 | 12594.83 | 19154.65 | 32201.63 | 23544.57 |

| **Table S3.** Primers used for qRT-PCR analysis of tsRNAs and mRNA levels | | |
| --- | --- | --- |
| Name | Primer sequence 5’-3’ | Product size (bp) |
| U6 | F:5’GCTTCGGCAGCACATATACTAAAAT3’  R:5’CGCTTCACGAATTTGCGTGTCAT3’ | 89 |
| tRF-Ala-AGC-057 | F:5’ AGTCCGACGATCTCAATCCCC 3'  R:5’ CTTCCGATCTTGGTGGAGGTG 3’ | 44 |
| tRF-Thr-AGT-019 | F:5’ AGTCCGACGATCTCCCACAT 3'  R:5’ GTGTGCTCTTCCGATCTGCT 3’ | 45 |
| tRF-Glu-TTC-026 | F:5’ CTACAGTCCGACGATCTCGAC 3'  R:5’ CTTCCGATCTTGGTTCCCAC 3’ | 48 |
| tRF-Gly-GCC-012 | F:5’ AGTCCGACGATCTCGATTCCC 3'  R:5’ TTCCGATCTTGGTGCATTGG 3’ | 43 |
| tRF-Thr-AGT-019 | F:5’ ACAGTCCGACGATCTCGAATC 3'  R:5’ CTTCCGATCTTGGAGGCACC 3’ | 46 |
| tRF-Gly-CCC-043 | F:5’ TCTACAGTCCGACGATCTCGAT 3'  R:5’ TCTTCCGATCTTGGTGCGC 3’ | 50 |
| GAPDH | F:5’ AATGGGCAGCCGTTAGGAAA 3’  R:5’ GCGCCCAATACGACCAAATC 3’ | 168 |
| iNOS | F:5’ GCCAAGCTGAAATTGAATGAGGA 3’  R:5’ TTCTGTGCCGGCAGCTTTAAC 3’ | 186 |
| CCL17 | F:5’ TGAGGACTGCTCCAGGGATG 3’  R:5’ AACGGTGGAGGTCCCAGGTA 3’ | 182 |
| HLA-DR | F:5’ AGAGCCTGGGTTTGCAGAGA 3’  R:5’ CCTGACTTCAATGCTGCCTG 3’ | 199 |
| IL-1β | F:5’ CAGAAGTACCTGAGCTCGCC 3’  R:5’ TGAAGCCCTTGCTGTAGTGG 3’ | 180 |
| Arg1 | F:5’ ACTTAAAGAACAAGAGTGTGATGTG 3’  R:5’ CATGGCCAGAGATGCTTCCA 3’ | 218 |
| CD163 | F:5’ TTGTAGCCACAGCAGGGATG 3’  R:5’ GGGGTAGAAAGGGCAACTCC 3’ | 201 |
| TNF-α | F:5’CCGAGTCTGGGCAGGTCTA 3’  F:5’CGAAGTGGTGGTCTTGTTGC 3’ | 201 |
| IL-6 | F:5’TGACCCAACCACAAATGC 3’  F:5’ CTGAGGTGCCCATGCTAC 3’ | 153 |

| **Table S4.** Mimics and inhibitor sequence | |
| --- | --- |
| **Name** | **Sequence (5’-3’)** |
| Control mimic | UUCUCCGAACGUGUCACGUTT |
| tRF-Glu-TTC-026 mimic | UCGACUCCCGGUGUGGGAACCA |
| Inhibitor control | CAGUACUUUUGUGUAGUACAA |
| tRF-Glu-TTC-026 inhibitor | UGGUUCCCACACCGGGAGUCGA |
